# Latent profile analysis approach to the relationship between daily ambulatory activity patterns and metabolic syndrome in middle-aged and elderly Japanese individuals: The Toon Health Study

**DOI:** 10.1265/ehpm.23-00110

**Published:** 2023-09-28

**Authors:** Naofumi Yamamoto, Koutatsu Maruyama, Isao Saito, Kiyohide Tomooka, Takeshi Tanigawa, Ryoichi Kawamura, Yasunori Takata, Haruhiko Osawa

**Affiliations:** 1 Faculty of Collaborative Regional Innovation, Ehime University, 3 Bunkyo-cho, Matsuyama, Ehime 790-8577, Japan; 2 Laboratory of Community Health and Nutrition, Special Course of Food and Health Science, Department of Bioscience, Graduate School of Agriculture, Ehime University, 3-5-7 Tarumi, Matsuyama, Ehime 790-8566, Japan; 3 Department of Public Health and Epidemiology, Faculty of Medicine, Oita University, 1-1 Idaigaoka, Hasama-machi, Yufu City, Oita 879-5593, Japan; 4 Department of Public Health, Juntendo University Faculty of Medicine, 2-1-1 Hongo, Bunkyo-ku, Tokyo 113-8421, Japan; 5 Department of Public Health, Juntendo University Graduate School of Medicine, 2-1-1 Hongo, Bunkyo-ku, Tokyo 113-8421, Japan; 6 Department of Diabetes and Molecular Genetics, Ehime University Graduate School of Medicine, Shitsukawa, Toon, Ehime 791-0295, Japan

**Keywords:** Accelerometer, Physical activity, Latent profile analysis, Metabolic syndrome

## Abstract

**Background:**

This cross-sectional study aimed to identify the accumulation patterns of objectively measured ambulatory activity (AA) variables in the Japanese middle-aged and elderly individuals and examine the relationship of these derivative patterns with metabolic syndrome (MetS).

**Methods:**

A total of 1850 participants (66.1% women, mean age: 57.7 years) provided objectively assessed AA data using a uniaxial accelerometer. The number of steps, time accumulated in light-intensity AA (LIAA) and moderate-to-vigorous intensity AA (MVAA), and the ratio of MVAA to total AA (LIAA + MVAA) were calculated. Latent profile analysis was used to identify groups of participants based on their distinct AA patterns. Logistic regression models were used to assess the association of groups with MetS after adjusting for age, sex, alcohol intake, and cigarette smoking.

**Results:**

Four distinct groups were identified: Group A had few steps and low levels of LIAA and MVAA; group B had a certain number of steps and recommended level of MVAA but low level of LIAA; group C had a certain number or more of steps, high level of LIAA, and recommended level of MVAA; group D had an extremely high number of steps and high levels of both LIAA and MVAA. The multivariate-adjusted odds ratio (95% CI) for MetS in groups B, C, and D relative to group A were 0.857 (0.611–1.201), 0.679 (0.500–0.922), and 0.434 (0.259–0.730), respectively. Groups C and D had significantly lower odds ratio of MetS compared to group A.

**Conclusion:**

AA pattern involving a certain number or greater of steps accumulated through not only MVAA but also LIAA may help reduce the risk of MetS compared to inactive AA pattern.

## Introduction

Metabolic syndrome (MetS) is defined as a cluster of conditions including abdominal obesity, hypertension, dyslipidemia, and hyperglycemia that increases the risk of onset of various conditions, such as type 2 diabetes, cardiovascular disease, and cancer [1]. Globally, the prevalence of MetS is increasing [2], and in the Asia–Pacific region, more than 20% of adults are estimated to have MetS [3].

Physical activity (PA) is an important factor that can be modified to prevent MetS. Engaging in PA has also been indicated to be an important factor for long and healthy life in the recently reported “Lifelong Health Support 10” [4]. A systematic review of studies, on the relationship between objectively measured PA and MetS, reported that the total amount of PA and the time in PA intensities significantly and independently in correlation with MetS [5]. However, the PA variables are mutually related, and the PA in the daily life of individuals is the combination of these PA variables [6]. To the best of our knowledge, no study currently has examined the relationship between the PA pattern in the daily life of individuals formed through such PA variables and MetS. By taking the interaction of PA variables in the daily life environment into consideration, it will be possible to elucidate the type of PA pattern associated with MetS. This information is considerably useful in the field of public health, especially to examine strategies that involve promoting PA to prevent MetS.

Latent profile analysis identifies the interaction between input variables to create naturally occurring profiles, or typical patterns, of combinations of different variables in a heterogeneous population [7, 8]. In other words, latent profile analysis approach is useful to help enhance the understanding of how interactions—in particularly those that occur concomitantly between more than two variables—occur within-persons, and how these interactions are related in outcome variables [9, 10]. In recent years, latent profile analysis has been gaining attention in the field of PA as a useful statistical method to convert complex behavior into patterns [11–13].

Most PA in daily life is accounted for by ambulatory activity (AA) such as walking and running [14, 15]. AA is an easy PA to practice and considered the easiest method to pass daily life in an active manner [16]. However, because it is difficult to remember such relatively low-intensity and intermittently occurring PAs, self-reported AA measurement may not be reliable and valid [17]. Therefore, in this study, we focused on objectively measured AA in daily life, with the aim of identifying accumulation patterns of AA variables in Japanese middle-aged and elderly individuals and examining the relationship between these derivative patterns and MetS.

## Methods

### Study population

The present study was a cross-sectional study, conducted as part of the Toon Health Study, a prospective cohort study in Toon City, Ehime Prefecture, Japan. The Toon Health Study commenced in 2009 and aimed to characterize risk factors for cardiovascular disease for its prevention in a community setting. In the Toon Health Study, we recruited participants from approximately 22,000 residents in Toon City who were 30–79 years old using newspaper advertisements, posters, and invitations. In all, 2,032 men and women participated in the baseline survey, conducted from 2009 to 2012. Participants were excluded if they had missing AA data (refusal to wear accelerometers, n = 115; wearing accelerometers for less than the required time/days, n = 67). In total, 628 men and 1222 women were eligible for the analyses. The characteristics of participants are shown in Table 1.

**Table 1:** Characteristics of the study population

	N = 1850	

Women (n, %)	1222	66.1
Age (years)	57.7	(12.5)
Body mass index (kg/m^2^)	23.1	(3.3)
Workers (n, %)	1053	56.9

Waist circumference (cm)	83.3	(9.5)
Systolic blood pressure (mmHg)	126.2	(20.1)
Diastolic blood pressure (mmHg)	76.0	(11.9)
Fasting glucose (mg/dL)	94.0	(13.9)
High-density lipoprotein cholesterol (mg/dL)	61.0	(14.5)
Triglycerides (mg/dL)	105.6	(58.7)

Smokers (n, %)	154	8.3
Drinkers (n, %)	831	44.9

Abdominal obesity (n, %)	555	30.0
Hypertension (n, %)	931	50.3
Hyperglycemia (n, %)	174	9.4
Dyslipidemia (n, %)	580	31.4
Pre-MetS (n, %)	225	12.2
MetS (n, %)	232	12.5
MetS/Pre-MetS (n, %)	457	24.7

Steps (step/day)	8439	(3404)
LIAA (min/day)	69.6	(26.0)
MVAA (min/day)	21.4	(16.3)
MVAA/total AA (min/min)	0.22	(0.12)

Data presented as mean (standard deviation) or n, %

MetS, metabolic syndrome; LIAA, light-intensity ambulatory activity; MVAA, moderate-to-vigorous intensity ambulatory activity; AA, ambulatory activity

Written informed consent was given by all participants. The study protocol was approved by the Human Ethics Review Committees of Ehime University Graduate School of Medicine (approval number: 170511).

### Ambulatory activity

For the AA of participants, we evaluated the number of steps and period of activity according to intensity using a Lifecorder-Ex (LC, Suzuken Co., Ltd., Japan) with a uniaxial accelerometer [18]. This device detects acceleration signals ranging from 0.06 G to 1.94 G at 32 Hz. When the sensor detects three or more accelerations within 4 sec, such activity is deemed as PA and classified into the LC’s unique activity intensity level of 1–9. Furthermore, when the level of acceleration is <0.06 G, the movement intensity will be 0. Moreover, when acceleration of ≥0.06 G is detected, which does not correspond to activity intensities 1–9, it will be perceived as micromovement and obtain an activity intensity value of 0.5. As described above, each activity every 4 seconds will be classified and recorded into one of 11 levels (0, 0.5, and 1–9) of activity intensity. The activity intensity on the LC is reported to be closely approximated the metabolic equivalents (METs) when walking and running. Furthermore, on the basis of previous research, the LC’s activity intensity of 1–3 was defined as light-intensity AA (LIAA), and activity intensity of 4–9 was defined as moderate-to-vigorous intensity AA (MVAA) [18]. The high level of measurement accuracy of the number of steps on the LC has been previously elucidated [19, 20].

The participants were instructed to wear the LC for 7 days continuously from waking up until bedtime, except when sleeping and during activity in water (taking a bath and swimming). In the present study, an LC intensity of ≥0.5 for a total of 8 hours or more in 1 day, and for ≥4 days was defined as valid data [21] in which we calculated the mean number of steps, LIAA, MVAA, and total AA (LIAA + MVAA) per day for each individual.

### Metabolic syndrome components

Height was measured to the nearest 0.1 cm using a wall-mounted stadiometer. Body weight was measured to the nearest 0.1 kg using a digital scale without shoes. BMI was calculated from body weight and height (kg/m^2^). Waist circumference (WC) was measured three times, to the nearest 0.1 cm, using a calibrated measuring tape at the midpoint of the lower costal margin; the mean value was used in the analyses.

Blood pressure (BP) was measured twice using an automatic sphygmomanometer (BP-103iII; OMRON Colin Co., Tokyo, Japan) while participants were seated after a rest of at least 5 minutes. The mean of the two measurements was used for analysis.

Overnight fasting blood samples were drawn from the antecubital vein into vacuum tubes containing a serum separator gel (for glucose and blood chemistry). The serum tube was centrifuged immediately at 3,000 g for 15 minutes, and the separated serum was sent to the laboratory for analysis. Enzymatic methods were used to measure serum levels of total cholesterol and triglycerides (TG). Low-density lipoprotein cholesterol and high-density lipoprotein cholesterol (HDL-C) were measured using the direct homogeneous method. Lipid measurements were standardized using the CDC NHLBI Lipids Standardization Program provided by the Centers for Disease Control and Prevention (Atlanta, GA, USA). Serum glucose was measured by the hexokinase method (Sysmex, Kobe, Japan) with an automatic analyzer (7600-D; Hitachi Co., Tokyo, Japan).

The Japanese criteria for MetS were used to evaluate the prevalence of MetS and pre-MetS in this study [22]. Based on these criteria, to diagnose MetS, the participant must present with abdominal obesity (WC ≥85 cm in men and ≥90 cm in women) in addition to two or more of the other components. For the diagnosis of pre-MetS, the participant must have abdominal obesity and one of the other components, including (1) dyslipidemia [TG ≥150 mg/dL (≥1.7 mmol/L) and/or HDL-C level <40 mg/dL (<1.0 mmol/L) or specific treatment for these lipid abnormalities]; (2) BP ≥130/85 mmHg or on drug treatment; and (3) fasting glucose ≥110 mg/dL (≥6.1 mmol/L) or on drug treatment. Ministry of Health, Labor, and Welfare of Japan considers MetS and pre-MetS as important targets to reduce the risk of MetS-related diseases [23]. Therefore, in line with previous studies [24–26], we analyzed AA pattern for MetS and pre-MetS participants in a single group (MetS/pre-MetS).

### Self-reported physical activity and sedentary time

For interpretation of the identified AA patterns, domain-specific PA and sedentary time (ST) were assessed using the Japan Arteriosclerosis Longitudinal Study Physical Activity Questionnaire (JALSPAQ), which was previously validated [27]. The details of JALSPAQ were described in a previous study [27]. We calculated METs·hour per day for determining occupational (work-related), transportational (walking or bicycling to work or shopping), household, and leisure-time PAs. The METs·hour per day of leisure-time PA was calculated separately for exercise and nonexercise activities. Individuals who performed paid work 1 day or more per week were considered workers.

The JALSPAQ includes questions about sedentary behaviors in two domains (occupational and leisure-time activities). Overall ST was calculated as the sum of leisure time and occupational ST. The ST assessed using JALSPAQ exhibited significantly correlation with accelerometer-determined ST [28].

### Covariates

Using a self-administered questionnaire, we surveyed drinking and smoking habit. With regard to smoking, individuals who responded that they presently smoke tobacco were considered smokers, and with regard to drinking, individuals who consumed 1 g or more of alcohol per week were considered drinkers.

### Statistical analysis

Namely, four variables were used in the latent profile analysis, the number of steps, LIAA, MVAA, and the ratio of MVAA to total AA. In reference to previous research [12], we used the mean and standard deviation for these variables, which we converted into z scores for use in our analysis. In earlier research, to identify the characteristics of PA patterns, the absolute value as well as the relative value of PA variables were used [12]. Therefore, in the present study, in addition to three variables of AA measured using the LC (number of steps, LIAA, and MVAA), we calculated and used the ratio of MVAA to total AA, which is proportionate of MVAA to daily total AA. Steps are a basic unit of locomotion and provide an easy-to-understand metric of ambulation [29]. Therefore, we used steps as an index of daily amount of AA in the analysis. However, regarding the relative value of AA, the ratio of MVAA to number of steps is difficult to interpret due to the difference in unit, and therefore we used the ratio of MVAA to total AA, whose unit is min/min and easy to interpret.

To identify the latent profile, we implemented a continuous latent model with 2–5 profile solutions. To derive the optimal number of profiles, the Akaike Information Criterion (AIC), Bayesian Information Criterion (BIC), entropy, and group size of each profile was evaluated for each model from the two-profile model to a five-profile model. In addition, we performed a group interpretation [30] and selected the final model. Lower the AIC and BIC values, better the goodness-of-fit of the model. For entropy, a value of 0–1 was given, and values ≥0.8 were considered to indicate good profile classification. Group size was based on ≥5% of participants belonging to each group of the total participant size [31]. For the latent profile analysis, we used Mplus (Version 8.5, Muthén & Muthén, Los Angeles, CA).

Categorical variables between AA patterns (groups) identified by latent profile analysis were compared using a chi-square test, and to compare continuous variables, a one-way analysis of variance was performed using the Bonferroni method for multiple comparisons. First, to confirm the relationship between AA variables and MetS, we conducted a logistic regression analysis using MetS as a dependent variable and each AA variable (quartiles of steps, LIAA, MVAA, and total AA) as an independent variable. Next to examine the relationship between AA pattern and MetS, we conducted a logistic regression analysis using MetS as a dependent variable and AA patterns (four groups) as independent variables. On the basis of the group with the lowest activity level, we calculated the odds ratio and the 95% confidence interval (95% CI) of the other groups. As confounder variables, we incorporated sex (men/women), age (continuous variable), smoking habit (with/without), and drinking habit (with/without) into the model. MetS was analyzed for individuals who corresponded to MetS and pre-MetS and were defined as cases, and individuals who did not correspond to either as noncases. The statistical analyses were performed using software SPSS version 25.0 (IBM Corp, Armonk, NY, USA), and *p* < 0.05 was considered statistically significant (two-tailed test).

## Results

The fit statistic of the latent profile model is presented in Table 2. In the two-to-five profile models examined, the AIC and BIC decreased as the profile number increased. In all of the models, a value of ≥0.8 was obtained for entropy. Entropy was greatest in the two-profile model; however, entropy decreased in the three-profile model, and increased in the four-profile model. With regard to group size, the five-profile model included groups that had fewer participants than the recommended cutoff value (5%). Based on these fit statistics and interpretability of the later-mentioned groups, in the present study, we used the four-profile model as the optimal model.

**Table 2:** Fit indices of 2- to 5-profile latent models

	**2 Profiles**	**3 Profiles**	**4 Profiles**	**5 Profiles**
AIC	18635	17960	16886	16160
BIC	18707	17790	1713	16314
Entropy	0.899	0.817	0.862	0.855
Group size	1465/385	983/651/216	1047/261/ 418/124	710/674/212/ 190/64

AIC, Akaike information criterion; BIC, Bayesian information criteria

The PA characteristics of the four groups classified by latent profile analysis are presented in Table 3. The number of steps was significantly high in descending order of groups A, B, C, and D. There was no significant difference observed in LIAA between groups A and B, and between groups C and D. MVAA was significantly high in descending order of groups A, B, C, and D. Occupational PA in group C was significantly higher than that in groups A and B, and group D had significantly higher occupational PA than group A. Transportation PA in group C was significantly higher than that in group A. Household PA in groups A and C was significantly higher than that in groups B and D. Exercise level during leisure time was the highest in Group D, and there was no significant difference in the exercise levels between groups A and C. ST in Group C was significantly shorter than that in other groups.

**Table 3:** Ambulatory activity and domain-specific physical activity variables among the identified distinct groups

	**Group-A (n = 1047)**		**Group-B (n = 261)**		**Group-C (n = 418)**		**Group-D (n = 124)**		**p**

	**mean**	**s.d.**	**mean**	**s.d.**	**mean**	**s.d.**	**mean**	**s.d.**	
Step (step/day)	6217	1625^b,c,d^	9507	1720^a,c,d^	11166	1907^a,b,d^	15755	3077^a,b,c^	<0.001
LIAA (min/day)	57.3	15.6^c,d^	58.5	14.1^c,d^	99.5	20.1^a,b^	96.8	31.5^a,b^	<0.001
MVAA (min/day)	11.6	5.9^b,c,d^	36.7	8.8^a,c,d^	24.4	7.7^a,b,d^	62.0	17.3^a,b,c^	<0.001
MVAA/total AA (min/min)	0.17	0.08^b,c,d^	0.39	0.08^a,c^	0.20	0.06^a,b,d^	0.40	0.11^a,c^	<0.001

Occupational PA (METs·h/day)	4.6	5.8^c,d^	5.4	6.1^c^	8.1	8.0^a,b^	6.5	7.5^a^	<0.001
Transportational PA (METs·h/day)	2.1	2.3^c^	2.3	2.4	2.5	3.3^a^	2.3	2.1	0.030
Household PA (METs·h/day)	6.4	5.7^b,d^	5.3	5.4^a,c^	6.7	5.4^b,d^	4.4	4.1^a,c^	<0.001
Exercise leisure activity (METs·h/day)	0.8	2.1^b,d^	2.1	1.9^a,c,d^	1.2	1.7^b,d^	2.9	3.0^a,b,c^	<0.001
Nonexercise leisure activity (METs·h/day)	0.8	2.1	0.7	1.7	0.8	2.1	0.6	1.1	0.798
Sedentary time (h/day)	10.8	3.1^c^	10.6	3.1^c^	9.6	3.1^a,b,d^	10.9	2.7^c^	<0.001

^a^p < 0.05 vs Group-A; ^b^p < 0.05 vs Group-B; ^c^p < 0.05 vs Group-C; ^d^p < 0.05 vs Group-D

LIAA, light-intensity ambulatory activity; MVAA, moderate-to-vigorous intensity ambulatory activity; AA, ambulatory activity; PA, physical activity

The characteristics of MetS-related indices between the four groups are presented in Table 4. WC and TG tended to be low in descending order of groups A, B, C, and D; whereas, HDL-C tended to be high in descending order of groups A, B, C, and D.

**Table 4:** Characteristics of participants in the identified distinct groups

	**Group-A** **(n = 1047)**		**Group-B** **(n = 261)**		**Group-C** **(n = 418)**		**Group-D** **(n = 124)**		**p**
Women (n, %)	720	67.8	155	59.4	290	69.4	67	54.0	0.001
Age (years)	58.5	(12.9)	57.4	(12.4)	56.0	(11.7)	57.8	(11.9)	0.007
Body mass index (kg/m^2^)	23.1	(3.4)	23.3	(3.0)	22.9	(3.2)	23.0	(3.2)	0.367
Workers (n, %)	545	52.1	149	57.1	287	68.7	72	58.1	<0.001

Waist circumference (cm)	84.0	(9.4)	83.3	(8.5)	82.3	(9.1)	80.8	(12.0)	<0.001
Systolic blood pressure (mmHg)	126.1	(20.4)	128.0	(20.2)	125.0	(19.2)	128.0	(20.5)	0.193
Diastolic blood pressure (mmHg)	75.8	(11.8)	77.3	(11.8)	75.5	(11.7)	77.1	(12.4)	0.182
Fasting glucose (mg/dL)	94.1	(14.8)	94.9	(14.3)	92.5	(10.6)	96.0	(15.2)	0.033
High-density lipoprotein cholesterol (mg/dL)	59.5	(14.5)	60.7	(14.5)	63.2	(14.1)	66.7	(13.9)	<0.001
Triglycerides (mg/dL)	109.5	(62.6)	105.5	(52.0)	101.3	(55.3)	87.9	(42.1)	<0.001

Smokers (n, %)	101	9.6	16	6.1	32	7.7	5	4.0	0.063
Drinkers (n, %)	452	43.2	104	39.8	208	49.8	67	54.0	0.007

Abdominal obesity (n, %)	333	31.8	87	33.3	104	24.9	31	25.0	0.021
Hypertension (n, %)	543	51.9	139	53.3	184	44.0	65	52.4	0.033
Hyperglycemia (n, %)	101	9.6	24	9.2	31	7.4	18	14.5	0.120
Dyslipidemia (n, %)	343	32.8	90	34.5	118	28.2	29	23.4	0.053
Pre-MetS (n, %)	139	13.3	33	12.6	42	10.0	11	8.9	0.233
MetS (n, %)	147	14.0	36	13.8	38	9.1	11	8.9	0.035
MetS/Pre-MetS (n, %)	286	27.3	69	26.4	80	19.1	22	17.7	0.002

Data presented as mean (standard deviation) or n, %

The crude and multivariate-adjusted odds ratios for MetS according to quartiles of AA variables are presented in Table 5. A linear relationship was found between all AA variables and MetS (steps, p for trend ≤ 0.001; LIAA, p for trend ≤ 0.001; MVAA, p for trend = 0.002; total AA, p for trend ≤ 0.001). No significant interactions were detected in the examination of the associations between each AA variables, sex, and MetS (steps, p for interaction = 0.939; LIAA, p for interaction = 0.722; MVAA, p for interaction = 0.871; total AA, p for interaction = 0.939).

**Table 5:** Odds ratio and 95% confidence intervals for metabolic syndrome in different amburatory activity levels (quartiles)

	**n**	**Case**	**Crude OR (95% CI)**		**Maltivariable adjusted OR^a^ (95% CI)**	
Steps (steps/day)						
Q1 (<6088)	464	161	1.000	(reference)	1.000	(reference)
Q2 (6088–7969)	461	106	0.562	(0.421–0.750)	0.627	(0.456–0.863)
Q3 (7970–10257)	462	102	0.533	(0.398–0.714)	0.598	(0.433–0.825)
Q4 (>10257)	463	88	0.442	(0.327–0.597)	0.446	(0.320–0.622)
P for trend			p < 0.001		p < 0.001	

LIAA (min/day)						
Q1 (<51.2)	459	152	1.000	(reference)	1.000	(reference)
Q2 (51.2–65.8)	466	125	0.740	(0.558–0.982)	0.772	(0.565–1.054)
Q3 (65.9–83.9)	462	95	0.523	(0.388–0.704)	0.598	(0.431–0.830)
Q4 (>84.0)	463	85	0.454	(0.335–0.616)	0.470	(0.336–0.656)
P for trend			p < 0.001		p < 0.001	

MVAA (min/day)						
Q1 (<10.2)	456	154	1.000	(reference)	1.000	(reference)
Q2 (10.3–17.4)	469	104	0.559	(0.417–0.748)	0.660	(0.477–0.914)
Q3 (17.5–27.8)	463	94	0.500	(0.371–0.673)	0.659	(0.471–0.922)
Q4 (>27.9)	462	105	0.577	(0.431–0.772)	0.582	(0.419–0.808)
P for trend			p < 0.001		p = 0.002	

Total AA (min/day)						
Q1 (<66.1)	463	156	1.000	(reference)	1.000	(reference)
Q2 (66.2–85.9)	463	117	0.665	(0.501–0.885)	0.728	(0.531–0.998)
Q3 (86.0–110.6)	462	96	0.516	(0.384–0.694)	0.556	(0.401–0.770)
Q4 (>110.6)	462	88	0.463	(0.342–0.626)	0.455	(0.326–0.635)
P for trend			p < 0.001		p < 0.001	

^a^Adjusted for sex (men, women), age (continuous variable), smoking habit (with/without), and drinking habit (with/without).

LIAA, light-intensity ambulatory activity; MVAA, moderate-to-vigorous-intensity ambulatory activity; AA, ambulatory activity

The crude odds ratio of the four groups to MetS and multivariate-adjusted odds ratios are presented in Table 6. The multivariate-adjusted odds ratio of groups B, C, and D to groups A (95% CI) were 0.857 (0.611–1.201), 0.679 (0.500–0.922), and 0.434 (0.259–0.730), respectively, with a significantly lower value obtained for groups C and D. As a sensitivity analysis, we calculated the odds ratios of the four groups to components of MetS (abdominal obesity, hypertension, hyperglycemia, and dyslipidemia). The odds ratio of the groups for the pertinence of abdominal obesity tended to be the same as the odds ratio for MetS. Table 7 shows the results of the analysis by sex. In men, the same trend of the analysis of all participants was observed. No definite trend in the odds ratio was observed in women, probably due to fewer cases in women than in men.

**Table 6:** Odds ratio and 95% confidence intervals for metabolic syndrome for the identified distinct groups

	**n**	**Case**	**Crude OR (95% CI)**		**Maltivariable adjusted ** **OR^a^ (95% CI)**	
MetS/Pre-MetS						
Group-A	1047	286	1.000	(reference)	1.000	(reference)
Group-B	261	69	0.956	(0.704–1.300)	0.857	(0.611–1.201)
Group-C	418	80	0.630	(0.476–0.832)	0.679	(0.500–0.922)
Group-D	124	22	0.574	(0.355–0.928)	0.434	(0.259–0.730)

Abdominal obesity						
Group-A	1047	333	1.000	(reference)	1.000	(reference)
Group-B	261	87	1.072	(0.804–1.430)	0.953	(0.691–1.314)
Group-C	418	104	0.710	(0.549–0.918)	0.739	(0.556–0.983)
Group-D	124	31	0.715	(0.466–1.095)	0.528	(0.330–0.843)

Hypertension						
Group-A	1047	543	1.000	(reference)	1.000	(reference)
Group-B	261	139	1.058	(0.806–1.388)	1.142	(0.836–1.558)
Group-C	418	184	0.730	(0.581–0.917)	0.855	(0.661–1.107)
Group-D	124	65	1.023	(0.704–1.484)	1.007	(0.660–1.535)

Hyperglycemia						
Group-A	1047	101	1.000	(reference)	1.000	(reference)
Group-B	261	24	0.948	(0.594–1.513)	1.013	(0.625–1.643)
Group-C	418	31	0.750	(0.493–1.141)	0.958	(0.619–1.483)
Group-D	124	18	1.591	(0.927–2.730)	1.703	(0.964–3.009)

Dyslipidemia						
Group-A	1047	343	1.000	(reference)	1.000	(reference)
Group-B	261	90	1.080	(0.812–1.438)	1.099	(0.815–1.483)
Group-C	418	118	0.807	(0.629–1.036)	0.953	(0.735–1.237)
Group-D	124	29	0.627	(0.405–0.968)	0.636	(0.405–1.000)

^a^Adjusted for sex (men, women), age (continuous variable), smoking habit (with/without), and drinking habit (with/without).

**Table 7:** Sex-stratified odds ratios and 95% confidence intervals for metabolic syndrome for the identified distinct groups

	**n**	**Case**	**Crude OR (95% CI)**		**Maltivariable adjusted OR^a^ (95% CI)**	
Men						
Group-A	337	176	1.000	(reference)	1.000	(reference)
Group-B	106	51	0.864	(0.574–1.300)	0.906	(0.581–1.413)
Group-C	128	52	0.595	(0.386–0.916)	0.658	(0.434–0.998)
Group-D	57	15	0.322	(0.175–0.593)	0.344	(0.182–0.650)

Women						
Group-A	710	110	1.000	(reference)	1.000	(reference)
Group-B	155	18	0.797	(0.499–1.273)	0.696	(0.403–1.203)
Group-C	290	28	0.576	(0.363–0.913)	0.697	(0.443–1.096)
Group-D	67	7	0.745	(0.360–1.541)	0.660	(0.289–1.505)

^a^Adjusted for age (continuous variable), smoking habit (with/without), and drinking habit (with/without).

## Discussion

In the present study, we adopted the latent model with four-profile solutions as the model with the best fit. Group A had few steps and a low level of MVAA, and was thought to be the profile that reflected insufficient PA pattern. In the review by Macniven et al. [32], 62% (interquartile range: 40%–85%) of adults in the Asia–Pacific region are estimated to be individuals with sufficient PA (that is, the value obtained upon subtracting these values from 100% gives the estimated rate of individuals with insufficient PA). In the present study, the group size of group A was 56.6%, which was the approximately the category reported in previous research [32]. Compared to group A, group B had significantly more steps and a significantly higher level of MVAA; however, there was no significant difference observed in terms of LIAA. Group C had a lower level of MVAA than group B; however, compared to group A, the number of steps, and level of LIAA and MVAA were significantly higher. In other words, this can be interpreted to indicate that group B is a group that accumulates a certain number of steps [33] primarily by MVAA; whereas, group C is a group that accumulates a certain number or more of steps [33] by both LIAA and MVAA. In addition, in groups B and C, the mean amount of MVAA per week was 256.9 and 170.8 minutes, respectively, which achieved the recommended amount in the PA guidelines [34]. Generally, it is thought that LIAA is primarily accumulated through daily life activities, such as housework and work (nonexercise activity), while MVAA is accumulated through each PA that intentionally occurs [35, 36]. Therefore, it is inferred that group B is a group with PA pattern called “active couch potato” [37], which has sufficient moderate-to-vigorous intensity PA time but also long time with little energy expenditure per unit of time (e.g., sedentary behavior) in daily life. This inference may be supported by the domain-specific PA data, which showed that only exercise leisure-time activity in group B was higher than that in group A, and household PA in group B has lower than that in group A. Group C is a group with PA pattern expressed as “move more and sit less” [38] proposed by the American PA guidelines. This inference may also be supported by the domain-specific PA data, which showed that occupational, transportational, and household PAs in group C were the highest among all groups, and ST in Group C was the lowest. Group D had an extremely high number of steps and LIAA and MVAA levels, and domain-specific PA data showed that both nonexercise PA (occupational PA) and exercise PA was high in this group. As group D was thought to have a very high level of PA, this group was referred to as “Busy Bee” in previous reports [39, 40]. The characteristics of each profile could be interpreted according to previous findings as mentioned above.

With regard to the odds ratio for MetS, in the present study there was no significant difference observed between groups A and B. Kudo et al. [26] compared MetS-related factors of the “group with low daily PA levels and without exercise habits” against the “group with a low daily PA levels with an exercise habit” in Japanese middle-aged and elderly individuals, and reported that there was no significant difference observed between the two groups. The results of the present study are supported by the reports by Kudo et al. [26]. Lindsay et al. [41], which examined the relationship between the amount and intensity of PA evaluated objectively and body fat evaluated using dual-energy X-ray absorptiometry, and reported that the amount of PA (total daily energy expenditure by PA) showed a strong inverse correlation with body fat. Furthermore, it has been reported that the time of light-intensity PA is a major factor to determine the level of energy expenditure via PA [41]. While the number of steps is an objective indicator of PA volume in daily life, the correlation with the level of energy expenditure by PA is not necessarily high [42–44]. From these findings we can infer that although group B accumulates a certain number of steps, it might not be a group with considerably higher level of energy expenditure by PA than group A. Therefore, the AA pattern of group B does not act to prevent obesity, which is a central component of MetS, and as a result, it is considered possible that a significant difference was not observed between group A and the prevalence of MetS.

On the other hand, the odds ratio for MetS in group C was significantly lower than group A. The total AA of group C was approximately 60 minutes longer than that of group A, and approximately 30 minutes longer than that of group B. In recent years, focus has been drawn to light-intensity PA and its impact on health, and it has been reported that independently of moderate-to-vigorous intensity PA, light-intensity PA shows an inverse correlation with cardiometabolic risk factors such as WC and TG [45]. The total AA of group C is accumulated for relatively long time through the accumulation of LIAA, in addition to achieving a certain level of MVAA [34]. It is inferred that through these synergistic effects, a significantly low odds ratio for MetS was obtained.

The odds ratio of group D for MetS was found to be lower than that of group C. While there was no significant difference in LIAA observed between groups D and C; group D was found to have significantly longer MVAA by approximately 40 minutes. In reports from Park et al. [46] of Japanese elderly and Kawakami et al. [25] of Japanese adults, a negative dose-response relationship has been found between moderate-to-high intensity PA and MetS. Our variable-centered analysis also revealed a liner relationship between MVAA and MetS (Table 5). The results of this study are supported by those of previous studies.

To the best of our knowledge, the present study is the first study to identify the AA pattern in daily life that occurs naturally in the Japanese population, and to examine the relationship with MetS. In the present study, we believe that through person-centered analysis of findings obtained by variable-centered analysis to date, we were able to provide additional useful information to be implemented in public health activities to prevent MetS. AA accounts for a lot of PA in daily life, and we believe that AA is the easiest method to make daily life active. Therefore, we believe that the findings of the present study provide useful information to examine PA to prevent MetS. However, the present study has several limitations. First, limitations owing to the measurement principle of the accelerometer used in the present study. The LC used in the present study is an activity monitor designed with the expectation of detecting the level of acceleration associated with vertical vibrations when walking and running [47]. A high level of accuracy with its measurement has been confirmed in earlier research, and as a method of measuring AA in daily life, it is highly valid and reliable [18]. On the other hand, the sensor on the device does not have the resolution required to sense slight movements such as activities of daily living including sedentary behavior. Furthermore, the LC does not have an algorithm to distinguish activities other than walking and running. In recent years, not only AA but also nonlocomotive PA and sedentary behavior has been recognized as important factors to affect health outcomes [34]. Therefore, additional tests should be performed in future such as identifying PA patterns of Japanese people using an objectively method that is capable of measuring various PA variables in daily life [48] and examining the relationship between these patterns and MetS. In doing so, we believe that it will enable the examination of more detailed measures to prevent MetS through PA. Second, the participants of the present study are volunteers, and it is possible that there is a selection bias. The number of steps of the subjects of the present study was somewhat higher than the average for both men and women Japanese people reported in the Japanese National Health and Nutrition Survey [49]. Therefore, the results of this study may not necessarily be applied to the general middle-aged and elderly population. Third, it has been reported that PA is influenced by the built environment [50, 51]. Thus, although this study was conducted on rural area residents, further study is warranted to be conducted on residents from other areas. Fourth, in women, groups B, C, and D showed lower odds ratio for MetS than group A, but the difference was not statistically significant, possibly due to smaller number of cases in women than men. Further studies are needed to use large sample size to clarify whether or not differences in the association between AA patterns and MetS are observed in men and women. Fifth, in this study, we were unable to adjust other confounding factors associated with MetS, such as food and snacks [2]. Finally, the present study is a cross-sectional study, and therefore, we cannot comment on the causal relationship between AA patterns and MetS. To correctly estimate the causal relationship and the causal effect between AA pattern and MetS, a longitudinal study should be performed in future.

## Conclusion

In the present study, we identified AA patterns in the daily life of middle-aged and elderly Japanese people, and examined the relationship with MetS. AA patterns of middle-aged and elderly Japanese people can be classified into four patterns, and we found that in the group with the AA pattern such as that in which a certain number or greater of steps is accumulated through the accumulation of not only MVAA but LIAA time also, the frequency of MetS was significantly lower than the group with an inactive AA pattern.

## Abbreviations

AA: Ambulatory activity
AIC: Akaike Information Criterion
BIC: Bayesian Information Criterion
BP: Blood pressure
HDL-C: high-density lipoprotein cholesterol
JALSPAQ: Japan Arteriosclerosis Longitudinal Study Physical Activity Questionnaire
LC: Lifecorder
LIAA: light-intensity ambulatory activity
MetS: Metabolic syndrome
METs: Metabolic equivalents
MVAA: Moderate-to-vigorous intensity ambulatory activity
PA: Physical activity
ST: Sedentary time
TG: Triglycerides
WC: Waist circumference
95% CI: 95% confidence interval

## Declarations

### Availability of data and material

Available upon a reasonable request.
